# Neighborhood safety, fall indices, physical activity level and social participation restrictions from a population of community-dwelling older adults in Nsukka, Enugu State, Nigeria

**DOI:** 10.1186/s12877-023-04059-x

**Published:** 2023-06-09

**Authors:** Christopher Olusanjo Akosile, Nnaemeka Pascal Ngwu, Uchenna Prosper Okonkwo, Ifeoma Uchenna Onwuakagba, Emmanuel Chiebuka Okoye

**Affiliations:** Department of Medical Rehabilitation, Faculty of Health Sciences and Technology, Nnamdi Nnamdi Azikiwe University, Awka, Nigeria

**Keywords:** Neighborhood safety, Physical activity, Social participation restriction, Older adults, Fall prevalence, Fall efficacy, Fall risk

## Abstract

**Background:**

Restriction in physical activity (PA) and social participation restriction (PR) can be heightened in the presence of fear of fall (FOF), fall experience, and perceived unsafe neighborhood, particularly among older adults. Despite the enormous benefits of social participation and physical activity, many older adults remain vulnerable to participation restriction and this probably accounts for a significant proportion of health challenges for older adults.

**Objective:**

This study investigated the relationship between neighborhood safety (NS), fall indices, physical activity, and social participation restriction among older adults from selected communities in Nsukka, Enugu state, Nigeria.

**Methods:**

This was a cross-sectional survey of 170 recruited via consecutive non-probability sampling techniques. Socio-demographic variables, co-morbidities, and fall prevalence were obtained using a self-administered questionnaire. The study instruments include the PA neighborhood environment scale – Nigeria (PANES-N), PA scale for elderly (PASE), Participation scale (PS), Modified fall efficacy scale (MFES), and Fall risk assessment tool (FRAT) and fall indices.

**Statistical analysis:**

Descriptive statistics of mean and standard deviations, frequency counts, and percentages were used to analyze the socio-demographic variables, and Inferential statistics of Spearman rank order correlation were used to determine the relationship among the neighborhood safety, fall indices, physical activity level, and participation restrictions.

**Results:**

PR has a negative relationship with NS (*r* = -0.19, *p*- 0.01), and fall efficacy (*r* = -0.52, *p*- 0.001). However, PR has a positive relationship with fall risk (*r* = 0.36, *p* = 0.001).

**Conclusion:**

Participation restriction is negatively correlated with neighborhood safety, fall efficacy, and PA. The PR has a positive relationship with fall risk (FR).

## Introduction

Aging is a natural and unavoidable process of life. It is typically associated with chronic health disorders among the older population. This negatively impacts their physical activity, social participation, and ability to live independently [1–3]. As aging progresses, structural and functional deterioration occurs due to physiological changes that affect various cells, tissues, organs, and systems which can collectively impact activities of daily living (ADL) and general well-being [3]. Age-related morbidities and the need for older adults to have adequate care call for enhanced attention among healthcare professionals. Amongst these is the need for increased participation in physical activity among older adults as a means to delay, prevent and manage many chronic health conditions associated with aging [4]. Regular physical activity participation contributes to improved physical and mental health and prevents diseases among older adults [5–7]. Lack of physical activity among older adults has been linked to several factors including health status (e.g. chronic health problems and pain), environmental factors (e.g. accessibility to exercise facilities and neighborhood safety), psychological issues (e.g. self-commitment, negative perception of exercise outcome) and demographics (e.g. education level and age group). This has led to an increase in deaths related to non-communicable diseases [8]. Even though the benefits of physical activity are well known, many older adults remain sedentary [4]. So there is a need to investigate the factors that influence physical activity participation in older adults. The role of neighborhood environments in encouraging individuals' adaptive behaviors, and promoting their health and well-being has become very important due to aging in our society.

The built environment is believed to play a large role in creating various opportunities for engaging in recreational physical activity [9]. A perceived or conceived low-safe neighborhood environment could surpass an older adult's physical ability to manage the demands of the environment, thereby predisposing them to hazards especially fall [10]. As the global population of older adults has increased progressively, fall is a major challenge as it affects health of a progressively increasing global population of older adults. Besides the fall itself, an important aspect to be emphasized in the older adult population is fall efficacy, as this can set up barriers in daily activities and cause a state of anxiety and even inhibition and/or restriction of these activities [11, 12]. Fall can reduce mobility and physical fitness, compromise lower limb muscles and the balance of the older adult, and consequently increase the risk of future falls. This way, fear of falling may be a predictor of falls and, consequently, of their negative repercussions for the older adult, including demands for individualized care [13]. The consequences of the fear of falling go beyond the clinical, psychological, social, and epidemiological spheres, and might include implications for the health, well-being, and quality of life (QoL) of the older adult [14]. Older adults with many limitations in their activities of daily living and insufficient accessibility in their neighborhood are more likely to fall, and such falls have serious consequences on the health of those older individuals [15]. There are important factors that can be addressed to reduce falls, such as balance, the habit of regular exercise, household ergonomics, the use of special shoes or other assistive devices, and medication, among others [15, 16].

Social participation is beneficial to an individual's health and well-being and important for maintaining a positive quality of life [17, 18]. Social participation involves not only sustaining relationships but also engaging in meaningful and purposeful activities. It also influences individuals' health and quality of life [17]. Encouraging and facilitating social participation in a community, especially among older adults can influence an individual's motivation to achieve and maintain physical and social activities. Advocating physical activity and social participation among older adults, especially in a safe neighborhood is an important part of the rehabilitation program in mitigating falls among this population.

A search through the literature has shown a dearth of published studies that investigated neighborhood safety, fall indices, physical activity level, and social participation restrictions among community-dwelling adults in a Nigerian population. However, a previous study in Nigeria reported that despite the enormous benefits of social participation and physical activity, many older adults remain vulnerable to participation and this probably accounts for debilitating health conditions among older adults [13]. Hence the current study aimed to investigate the relationship between neighborhood safety, fall indices, physical activity level, and social participation restrictions from a population of community-dwelling older adults in Nsukka, Enugu State, Nigeria.

## Methods

### Study design

Participants for this cross-sectional survey of 170 older adults (aged 65 years and above) were recruited via consecutive non-probability sampling techniques. Those recruited through convenient sampling provided information that lead to the recruitment of other participants who did not get the information earlier. Also, the four participating communities were selected by convenient sampling technique in Nsukka Local Government Area, Enugu State, Nigeria.

### Data collection procedure

The researchers approached the community, association, and religious leaders in the study population for an interactive session to explain the study to them. During the interactive session, the purpose and the procedures of the study were explained to them, and an opportunity was given to them to ask questions. Having understood the study and becoming convinced that the outcome of the study will be beneficial to their community they authorized the study to be conducted among their people. Following this approval by the critical stakeholders, an invitation was extended to would-be participants through the leaders of the communities and community associations, also by placing announcements and adverts in the community churches. The venues for data collection were the town or union halls and church halls in each community. The purpose of the study and the procedure for data collection was explained to each participant from whom informed consent was then obtained. Before giving their consent they were assured that all the information they would give would be confidential and that the procedure would cause them no harm. The instrument had two versions, the original version (English) and the translated version (Native language). The instrument of the study was back-translated (first from English to the Igbo language and then from the Igbo language to English) to ensure accuracy. The translation was effected by two linguistic experts, one in the English language, and one in the native language. This translation was reviewed by the research team to ensure the accuracy of the translated version. Those who were not literate to understand the content of the original version were given the translated version which was in the Igbo language. The prevalence and number of falls were obtained by asking the participant the number of times he/she had fallen in the past 6 months The instruments were administered to each participant by the researchers. The collated data was reviewed for missing data by the researchers and inputted into an excel spreadsheet by the statistician for data analysis.

### Description of the study instruments

#### Physical activity neighborhood environment scale in Nigeria (PANES-N)

An adapted version of the physical activity neighborhood environment scale in Nigeria (PANES-N) was used to assess the perception of neighborhood safety factors [19]. The 17-item PANES was originally developed by the international physical activity prevalence study and contained four Indicators of Crime and Traffic Safety [19]. The scores for the domain were summed up and divided by the number of items in the domain to have a domain score. The interpretation of the scoring when the response options are treated as continuous variables is that the higher the score (on a scale of 1 to 4) the more favorable the perception rating of the environment [20]. Scores closer to the maximum point of 4 (above 2) is deemed high score, while scores closer to the minimum score of 1 (below 2) is deemed low score.

#### Physical Activity Scale for the Elderly (PASE)

Physical activity level was assessed using the Physical Activity Scale for Elderly (PASE): Participation in leisure activities including walking outside the home, light sport/recreational activities, moderate sports/recreational activities, and strenuous sports/ recreational activities muscle strength/endurance, light housework, heavy housework/chores, home repairs, Lawn work/ yard care, outdoor gardening, caring for another person and work for pay or volunteer were recorded as never, seldom (1–2 days/week), sometimes (3–4 days/week), and often (5–7 days/week) [21]. The duration of the activities was categorized as less than 1 h, between 1 and 2 h, 2 to 4 h, or more than 4 h. Paid or unpaid work, other than work that involves mostly sitting activity, was recorded in total hours per week. Housework (light and heavy), lawn work/ yard care, home repair, outdoor gardening, and caring for others were recorded as yes/no [21].

PASE instrument average hours per day were calculated by multiplying the number of days of activities by the number of hours and then dividing by seven (i.e. 7 days of the week).

$$\mathrm{Mathematically\;stated},\mathrm{ average\;hour\;per\;day }= x=\frac{(\mathrm{days\;of\;activities\;x\;number\;of hours})}{7}$$

Then to get the PASE score, the average hour per day is now multiplied by the PASE weight. Mathematically stated as PASE score = Average hour/day x PASE weight for total activity performance (hours/day). The sum of all the average hours per day is divided by the total number of participants

$$\mathrm{Performance }= \frac{\mathrm{Sum\;of\;all\;the\;average\;hour}/\mathrm{day}}{\mathrm{total\;number\;of\;participants}}$$

Finally, the contribution PASE is calculated by multiplying the performance by the item weight.

Mathematically stated as contribution PASE = Performance x item weight.

This is for activities involving hours per day. The activities requiring YES or NO answers are calculated as:

- First, the options (YES or NO) values were multiplied directly by the PASE weight to get the PASE score. For total activity performance in percentage, it is calculated by dividing the total of all the participants that say 'YES' by the total number of all the participants and then multiplying by 100.
  $$\mathrm{Mathematically},\mathrm{ performance\;in\;\%\;age }= \frac{\mathrm{sum\;total\;of\;YES\;participants }}{\mathrm{total\;YES }+\mathrm{ NO\;participants }}\mathrm{ x }\;100\mathrm{\%}$$

Then the PASE contribution is calculated for those in percentage by dividing the performance by a hundred and multiplying by PASE weight,

$$\mathrm{PASE\;contribution }= \frac{\mathrm{performance}}{100}\mathrm{ x }\frac{\mathrm{item\;weight}}{1}$$

The PASE is a reliable and valid measure of physical activity in older adults and has been validated in older adults against two physical activity gold standards: doubly labeled water (r = 0.58) [21].

#### Prevalence of fall

Prevalence of falls was measured by subjectively asking the participants for a history and frequency of falls in the past six months and participants were grouped into categories from the information that were gotten as fallers and non-fallers for individuals that reported no fall, single fall and multiple/repeat falls respectively.

#### Modified Fall Efficacy Scale (MFES)

Fall efficacy was assessed using the Modified Fall Efficacy Scale (MFES). This is a 14-item questionnaire. Each item will be scored on the 10-point visual analog scale. '0' = not confident or not sure at all, 5' = fairly confident or fairly sure, '10' = completely confident or completely sure. The ranking is totaled (possible range of 0 – 140) and divided by 14 to get each subject's MFES score. Scores less than 8 indicate less confidence and low efficacy (fear of falling), and 8 or greater indicates more confidence and high efficacy (lack of fear).

#### Fall Risk Assessment Tool (FRAT)

The risk of falls was assessed using the Fall Risk Assessment Tool (FRAT). The FRAT has three sections: Part 1—fall risk status; Part 2 – risk factor checklist; and Part 3 – action plan. Fall risk status is categorized as low (5–11), medium (12–15), or high risk (16–20). Risk factors to be assessed include vision, mobility, transfers, behaviors, activities of daily living, environment, nutrition, and continence which can either be Y (yes) or N (No) [22].

#### Participation scale (PS)

Social participation restriction was assessed using the participation scale (PS): It assesses the restrictions in social participation. The participation scale is a test of 18 – items covering eight out of nine major life domains. The rating includes; no significant restriction (0 – 12), mild restriction (13 – 22), moderate restriction (23 – 32), severe restriction (33 – 52), and extreme restriction (53 – 90) [23].

### Data analysis

Obtained data were analyzed using IBM Statistical packages for the Social sciences (SPSS 26: SPSS Inc, Chicago, IL, USA). Descriptive statistics of frequency counts (for occupational categories and location), proportions, and percentages (for fallers and individuals with FOF) were used to determine the socio-demographic characteristics of the participants. The inferential statistics of Spearman rank order were used to determine the relationship between neighborhood safety, fall indices (fall risk and fall efficacy), physical activity level, and social participation restriction. The alpha level was set at 0.05.

## Results

One hundred and seventy community-dwelling older adults (51.8% males and 48.2% females) with a mean age of 70.77 ± 5.17 years participated in this study. The majority of the participants were from rural communities (81.2%), married (72.9%), and living with others (93.5%). A fair proportion of participants (40.7%) had at least a secondary level education and was still occupationally active (47.0%), while hypertension (28.2%) was the most prevalent health condition in the studied sample (Table 1).

**Table 1:** Socio-demographics frequency distribution

**Variable**	**Categories**	**Frequency**	**Percentage**
Gender	Male	88	51.8
	Female	82	48.2
Marital Status	Married	124	72.9
	Divorced/separated	7	4.1
	Widowed	39	22.9
Occupational status	Employed	15	8.8
	Self-employed	65	38.2
	Unemployed	56	32.9
	Retired	34	20.0
Educational level	None	21	12.4
	Primary	78	45.9
	Secondary	39	22.9
	Tertiary/higher	32	18.8
Locality/Environment	Urban	32	18.8
	Rural	138	81.2
Living arrangement	Living alone	11	6.5
	Living with others	159	93.5
Comorbidities	Hypertension	48	28.2
	Diabetes	31	18.2
	Arthritis	34	20.0
	Visual impairment	4	2.4
	Stroke	3	1.8
	Others	13	7.6

Table 2 shows that 100% of the participants and 86.5% of the older adults see their neighborhood as safe and having no restrictions respectively. These were suggestive of high physical activity levels and a perceived safe neighborhood environment among participants. The prevalence of falls in the studied population was 64 (37.6%). Twenty-four participants (14.1% of all those who had experienced at least a fall) had also sustained some injuries. All the participants regarded their neighborhood as safe and the risk of falls was generally low (0.6%). Despite these, however, a 41.2% prevalence of fear of falls (low fall efficacy) and a 13.5% prevalence of social participation restriction were recorded among participants. The single fallers and repeated fallers were 31(48.4%) and 33(51.6%) respectively.

**Table 2:** Participants categorization based on study scales

Variables	Variable Classification	Frequency Distribution	Percentage Distribution
**Neighborhood safety**	Safe	170	100.0
	Unsafe	0	0.0
**Participation restrictions**	No restrictions	147	86.5
	Mild restrictions	15	8.8
	Moderate restrictions	5	2.9
	Severe restrictions	3	1.8
**Fall risk**	Low risk	169	99.4
	Medium risk	1	0.6
**Fall efficacy**	Low efficacy (fearful)	70	41.2
	High efficacy (not fearful)	100	58.8
**Fall history in six months**	No	106	62.4
	Yes	64	37.6
**The proportion with or without injury**	Fall with injury	24	14.1
	The proportion of injury/fallers	24/64	37.5
**Fall category in proportion**	Single fallers	31/64	48.4
	Repeated fallers	33/64	51.6

Table 3 shows that walking outside the home with an activity performance of 0.43 h per day contributed 8.6 to the PASE score. Muscle strength/endurance had zero activity performance and zero contribution to the PASE score. Caring for another with 82% contributed 28.70 to PASE scores while working for pay or volunteering with 6% contributed 1.26 to the PASE score. The muscle strength /endurance with the activity weight of 30 contributed nothing to the PASE score. Light housework with an activity weight of 25 and heavy housework with an activity weight of 25 contributed 20.50 and 18.25 respectively to the total PASE score The whole variables contributed a total of 146.13 PASE scores.

**Table 3:** PASE activities, performance, weight score and contribution

PASE items	Type of activity	Activity weight	Activity performance	Contributions to PASE score
2	Walk outside home	20	0.43 Hours/day	8.6
3	Light sport/recreational activities	21	0.12 Hours/day	2.52
4	Moderate sports/ recreational activities	23	0.07 Hours/day	1.61
5	Strenuous sports/ recreational activities	23	0.01 Hours/day	0.23
6	Muscle strength/ endurance	30	0.00 Hours/day	0.00
7	Light housework	25	82%	20.50
8	Heavy housework/chores	25	77%	19.25
9a	Home repairs	30	70%	21.00
9b	Lawn work/ yard care	36	73%	26.28
9c	Outdoor gardening	20	78%	15.60
9d	Caring for another person	35	82%	28.70
10	Work for pay or volunteer	21	6%	1.26
**Total activity**			**PASE score**	**146.13**

Table 4 shows a spearman rank correlation between neighborhood safety, fall indices (fall efficacy and fall risk), physical activity level, and participation restrictions**.** Participation restriction was significantly correlated with neighborhood safety (*r* = -0.19, *p*- 0.01), fall efficacy (*r* = -0.52, *p*- 0.001*), and fall risk (*r* = 0.36, *p* = 0.001*). Aside from these, no significant correlation could be established among any other construct pair.

**Table 4:** Correlation matrix showing the relationship between neighborhood safety, fall indices (fall efficacy and fall risk), physical activity level and participation restrictions

Variables	NS	FE	FR	PA level	PR
NS		*r* = 0.13	*r* = 0.01	*r* = 0.04	*r* = -0.19
		*p* = 0.10	*p* = 0.91	*p* = 0.60	*p* = 0.01*
FE			*r* = 0.35	*r* = 0.12	*r* = -0.52
			*p* = < 0.001*	*p* = 0.12	*p* = < 0.001*
FR				*r* = -0.05	*r* = 0.36
				*p* = 0.54	*p* = < 0.001*
PA level					*r* = -0.09
					*p* = 0.24
PR					

Key:

^*^Correlation is significant at α level 0.05

*NS* Neighborhood safety, *FE* Fall efficacy, *FR* Fall risk, *PA level* Physical activity level, *PR* Participation restriction

## Discussion

This present study examined the levels of neighborhood safety, physical activity, fall indices (fall incidence, fall risk, and fall efficacy), and social participation restriction among a population of community-dwelling older adults in sub-urban Nigeria. Among the participants, the older male adults were more than older adult females. The marital status shows that 72.9% were married, 22.9% were widowed, and 4.1% were separated. The occupational status shows that 8.8% were still actively employed, 65% were self-employed, and 32.9% were unemployed. The educational status of the older adults revealed that 18.8% had a higher degree, 22.9% had a secondary certificate and 12.4% were not literate. The three common comorbidities common in the older adult population for this study were hypertension 28.5%, arthritis 20%, and Diabetes18.2%. Only 6.5% were living alone. Also, most of the participants were rural dwellers 81.2%.

The participants perceived their neighborhood as safe. This might be because the majority of the participants were rural dwellers and were living with either their children, grandchildren, or extended relatives. Also, it might be because they have gotten used to the environment coupled with a lack of diversified infrastructure prevalent in the urban environment which could create safety challenges. The finding that there was no significant correlation between neighborhood safety and overall physical activity level in the current study might relate to the report of the previous study that rural dwellers tend to be more physically active and equally had minimal participation restrictions when compared to urban areas where neighborhood safety issues are usually prevalent [20]. Despite the safe neighborhood and physical activity status of participants, high fall prevalence and low fall efficacy were reported. This might be attributed to the terrain, as this study was conducted during raining season, as well as the lack of planned infrastructure, bad roads, poor drainage system, and unattended domestic animals. This contrasts with the finding in urban areas reported by previous studies where neighborhood safety might be attributed to a high level of residential density, traffic, crime, and poor planning [20, 24]. Neighborhood safety seems not to be a major problem among rural-dwelling older adults in southeastern Nigeria as a similar study had revealed that older adults from selected communities in Nnewi, Anambra state described their neighborhood as safe [25].

Fall prevalence was high among participants despite their high physical activity level and occupationally active status. The prevalence might be influenced by environmental factors (such as rain), the nature of their roads, and neighborhood terrain as most of the fallers did not sustain injury and repeated fallers comprised some of the fall victims. Fall prevalence was assumed to be more among older participants as the frequency of falls increases with age and frailty level, this might be because thirty percent of people over 65 years of age, and 50% of those over 80 years of age fall each year and some older adults who fall once are two to three times as likely to fall again within a year.

The current study also revealed that the risk of falls was low among participants. This might be attributed to the understanding of fall prevention strategies by the respondents. This might, also, relate to the result of another study that identified low fall risk as a variety of personal strategies used by older adults to increase safety and navigate the perceived fall risks in their neighborhood [26]. Also, fall efficacy was low, suggesting a high prevalence of fear of falling among study participants. This might suggest that among the participants, individuals who have fear of falling could be repeated fallers, those that sustained fall-related injuries, and significantly older participants in the communities. The low fall efficacy might be an indicator of the post-fall syndrome as community-based studies have presumed that fear of falling might be a risk factor for future falls [27]. We think that the fall efficacy can be made worse among the study population if the older adults have seen those who have fallen before and sustained some bodily harm. This would likely make them develop a phobia of engaging in some physical or social activities. A significant correlation existed between fall efficacy and fall risk. This could be attributed to the preponderance of retired and occupationally less active study participants.

Participation restriction was recorded among some of the study participants. This might suggest that social participation among rural-dwelling older adults tends to decrease with age due to poor socio-economic status, such as income, education, occupation, morbidity and not having immediate family members around due to rural–urban migration. Participation restrictions were significantly correlated with neighborhood safety, fall risk, and fall efficacy among the participants. However, social participation restrictions might have contributed to the prevalence of falls and low fall efficacy among participants and were assumed to be more prevalent among repeated fallers, retired participants, and those living alone. A previous study reported that enhancing social participation in social activities could decrease frailty risk among middle-aged and older populations, especially in communicative activities, intellectually demanding/engaging activities, and community-organized physical activities [28].

In this present study, the safe neighborhood might have justified the high physical activity level among community-dwelling older adults. High fall prevalence and low fall efficacy might be due to environmental factors, seasonal variations, older participants, and post-fall syndrome, while participation restrictions might be due to social isolation among some participants. The results from this study showed that participants had low fall efficacy and high fall incidence. This might have contributed to a negative correlation between fall efficacy and participation restriction. A significant correlation also existed between fall efficacy and fall risk. Aside from these, no significant correlation was established among other variables. This might suggest that despite low fall efficacy, a significant proportion reported low fall risk which might be attributed to the instrument used in this study and other factors that have been previously highlighted.

Fear of falling can have different presentations among older adults as it defines the level of confidence in carrying out daily activities without falling. Also, less social participation might be due to the increasing prevalence of falls and low efficacy among community-dwelling older adults.

The current study shows that the participation restriction was negatively correlated with neighborhood safety (*r* = -0.19, *p*- 0.01). This was in agreement with an earlier study that stated that perceived neighborhood safety was a robust predictor of mobility limitation recovery [29]. The implication is that the higher the participation restriction the less the neighborhood safety. For example, living in neighborhoods with poor street conditions, heavy traffic, and excessive noise is associated with the onset of difficulty with movement-related activities (e.g. standing in place, lifting heavy objects, and climbing stairs) [30, 31]. Also, the fall efficacy was revealed to have a negative relationship with social restriction participation (*r* = -0.52, *p*- 0.001*). Older adults with low efficacy could be restrained from participation in social activities because of fear of falling. This finding is in line with the previous finding that observed that falls and the fear of falling are also significantly associated with a restriction in daily activities, mainly in those related to mobility, which may increase the subsequent risk of falling and of losing autonomy (post-fall syndrome) [32, 33]. The fall risk was found to have a positive relationship with the social participation restriction (*r* = 0.36, *p* = 0.001*). This implies that the presence of fall risk factors in both indoor and outdoor social activity could bring about participation restrictions. A previous study has highlighted social participation to be linked to frailty and falls risk, and social isolation to be a predictor of falls [34]. The longer individuals live, the greater their risk of developing age-related conditions, with the population aged 80 years and above increasing the most rapidly and representing individuals at the highest risk of falls and frailty [35].

### Summary of findings

A high prevalence of falls and low fall efficacy was recorded among these Nigerian community-dwelling elderly. Physical activity level was generally high and the neighborhood environment was considered generally safe. However, a considerable proportion has some form of participation restrictions. Inferentially, participation restriction was significantly correlated with neighborhood safety, fall efficacy, and fall risk. A significant correlation equally existed between fall risk and fall efficacy, while other variables could not establish a significant correlation. There is a need for routine physical and general health assessment as well as psychological counseling in these communities to address the issues of fall prevalence, low fall efficacy, and social participation restrictions.

## Recommendation

Future studies should examine the effect of socio-demographic characteristics on neighborhood safety, fall efficacy, social participation restriction, physical activity, and fall risk. Also, a randomized controlled study should be considered that will involve multicenter sites.

## Data Availability

The data is with the corresponding author and will be made available at a reasonable request.

## Abbreviations

PA: Physical activity
PR: Social participation restriction
NS: Neighborhood safety
PANES-N: PA neighborhood environment scale – Nigeria
PS: Participation scale
PASE: PA scale for elderly
FRAT: Fall risk assessment tool
ADL: Activities of daily living
Qol: Quality of life
MFES: Modified fall efficacy Scale
FOF: Fear of fall
